# Masticaticatory muscles characteristics in relation to adiposity and general muscular fitness: a population-based study

**DOI:** 10.1007/s10266-023-00785-1

**Published:** 2023-01-24

**Authors:** Peter Meisel, Amro Daboul, Robin Bülow, Michael Eremenko, Henry Völzke, Rainer Biffar, Thomas Kocher

**Affiliations:** 1 Department of Periodontology, Dental Clinics, School of Dentistry, University Medicine Greifswald, Fleischmann-Strasse 42, 17475 Greifswald, Germany; 2 Department of Prosthodontics, Gerodontolgy and Biomaterials, University Medicine Greifswald, Greifswald, Germany; 3 Institute for Radiology and Neuroradiology, University Medicine Greifswald, Greifswald, Germany; 4 Institute for Community Medicine, University Medicine Greifswald, Greifswald, Germany

**Keywords:** Aging, Obesity, Body fat, Teeth, Mastication, Hand grip strength

## Abstract

There is still considerable controversy surrounding the impact of mastication on obesity. The aim of this study was to identify the interplay between the masticatory muscles, teeth, and general muscular fitness and how they contribute to body adiposity in a general German population. This cross-sectional study included 616 participants (300 male, 316 female, age 31–93 years) from the population-based Study of Health in Pomerania. The cross-sectional areas of the masseter, medial and lateral pterygoid muscles were measured using magnetic resonance imaging (MRI), muscular fitness assessed by hand grip strength (HGS) and body fat distribution was measured by bioelectrical impedance analysis (BIA) and MRI. The overall prevalence of obesity was high in our cohort. The cross-sectional area of the masseter muscles was positively associated with the number of teeth, body mass index (BMI) and HGS, and negatively associated with the BIA-assessed body fat when adjusted for age, sex, teeth, and BMI. Especially the correlation was strong (*p* < 0.001). Analogous relationships were observed between the masseter, HGS and MRI-assessed subcutaneous fat. These associations were most pronounced with masseter, but also significant with both pterygoid muscles. Though the masticatory muscles were affected by the number of teeth, teeth had no impact on the relations between masseter muscle and adiposity. Physical fitness and masticatory performance are associated with body shape, controlled and directed by the relevant muscles.

## Introduction

The worldwide rampant obesity crisis is an increasing challenge facing public health systems. Obesity is the consequence of an imbalance between caloric intake and energy expenditure, modified by complex and interacting genetic, hormonal, environmental, and behavioural factors. Dietary intake starts with the oral processing of nourishments governed by mastication, which includes the number of functional teeth and the function of the chewing musculature. Whereas the association between tooth loss and obesity is ascertained knowledge [1, 2], the relationship between the functionality of mastication and obesity is controversial [3]. The main determinant of masticatory ability and efficiency is the synergy of teeth and chewing muscles. Thickness, volume or cross-sectional areas of masticatory muscles such as masseter and the pterygoids were mostly studied in relation to nutritional status or anthropometric characteristics. Besides age and sex differences, these muscles are strongly inter-related to bite force, number and positioning of teeth [1], and periodontal conditions [4]. Based on contemporary knowledge, tooth loss is accompanied by and concomitantly associated with weakened chewing musculature leading to obesity [3]. Furthermore, systemic characteristics such as skeletal musculature, physical fitness, and general health status show correlations with chewing ability. Decline in functional ability with advancing age eventually may be inevitable.

A possible impact of mastication on obesity has found broadened interest [3]. Compromised nutrition as a result of poor chewing ability can adversely affect systemic health, often primarily noticeable in fat distribution. Finely coordinated forces of teeth, tongue, chewing muscles together with neuromuscular and sensorial stimuli are important, under unfavourable conditions possibly affected by periodontal disease.

Close relationships between skeletal muscles in general and the chewing muscles lead to the assumption that mastication may be related to obesity as it is also known for sarcopenic states [5]. Masseter muscle representative for mastication is associated with body mass index (BMI) and also with skeletal muscle as characterized by measurements of hand grip strength [6]. In turn, skeletal muscles are weakened in states of overweight and obesity as manifested in sarcopenic obesity [7].

Insufficient mastication may lead to malnutrition [8] or to obesity [3, 9], according on whether the type of diet used is affected or mastication-induced behavioural changes influence food intake and processing [10, 11]. The relationship between masticatory muscles and skeletal musculature is important as both are correlated with obesity. A frequently used indicator of the state of the muscular system is hand grip strength (HGS), often used as a proxy for general health. It is also strongly associated with obesity [12].

The primary goal of this study was to identify possible relationships between body adiposity, masticatory muscles cross-sectional area, and muscular fitness. We aim to find out whether the relationship between masticatory muscles and obesity is merely a reflection of the general interaction of skeletal muscles with fat deposits or that the oral part of the musculature influences obesity by its own impact.

## Material and methods

### Study design

The baseline Study of Health in Pomerania (SHIP-START-0) comprised adult residents in the German area of West Pomerania [13]. Randomly drawn from local registries, 4,308 Caucasian subjects participated (1997–2001). The second follow-up was conducted 10 years later (SHIP-START-2) and was launched in 2008. It comprised 2333 subjects aged 30–90 years. Here, the participants of this latter study were cross-sectionally analysed. The final number of the study comprised 300 men and 316 women, respectively. Only those subjects of the original study participants were included who agreed to undergo a whole-body magnetic resonance imaging (MRI) and the head-neck MRI examination [14, 15]. Chewing muscle data, volumes of visceral and subcutaneous fat were assessed. Excluded were all those participants in which either of the two MRI measurements was lacking. Reporting was done in accordance with the STROBE guidelines. All participants gave their written informed consent and the study was approved by the ethics committee of Greifswald University.

### Oral and MRI measurements

Periodontal assessment included probing depth (PD), clinical attachment level (CAL), dental plaque, bleeding on probing, and the number of teeth. For a number of teeth all fully erupted natural teeth were assessed excluding the third molars. The periodontal examination was carried out on either the left- or right-side quadrants and the examination side was changed from participant to participant. CAL and PD were assessed at mesiobuccal, distobuccal, midbuccal and midlingual aspects on each selected tooth probe PCP 15, HuFriedy, Chicago IL).

Head-neck MRI scans were part of a whole-body examination assessed on a 1.5-T system (MAGNETOM® Avanto, Siemens Erlangen, Germany). MRI technique for scanning the masseter and pterygoid muscles and for analysing the MRI head images were described elsewhere [14]. The cross-sectional areas of the masseter, medial and lateral pterygoid muscles were registered in cm^2^ of both muscle sides and the maximum area of either side was used. Chewing muscle’s strength is closely related to its cross-sectional area as used in this study [16]. Standardized whole-body MRI measurements were taken covering the full abdomen [14] enabling quantification of subcutaneous and visceral fat volumes (liter) employing the automatic tissue analysis software ATLAS (Scientific Software Development, Berlin, Germany).

### Handgrip strength, anthropometry, and laboratory data

Lean body mass and body fat mass were measured by bioelectrical impedance analysis (BIA) using a multifrequency Nutriguard‐M device (Data Input, Pöcking, Germany). The electrodes were placed on the hand, wrist, ankle, and foot and test frequencies were measured according to the manufacturer's instructions [17].

HGS was measured by a handheld Smedley-type dynamometer (Scandidact, Denmark) left- and right-handed, with the maximum strength in kg of either hand for analyses [18]. Measurements of anthropometric data were taken using balance and height measuring devices (Soehnle, Murrhardt, Germany), body weight to the nearest 0.10 kg, height to the nearest cm, waist and hip circumferences to the nearest 0.5 cm.

Body mass index (BMI in kg/m^2^) was calculated, also waist circumference (WC) and waist-to-hip ratio (WHR). BMI categories were defined as 18.5–< 25 kg/m^2^, ≥ 25< 30, and ≥ 30, sex-specific WC risk thresholds were ≥ 88 and ≥ 102 cm for women and men, respectively. Glycated hemoglobin (HbA1c) and fibrinogen were measured by standard laboratory methods and high sensitivity CRP in serum by particle-enhanced immuno-nephelometry (Dade Behring Inc., Eschborn, Germany). Indicators of socio-demographic variables were obtained from the health-related interviews or the personal questionnaire.

### Statistical analyses

We used the software STATA 14.0 (Stata Corp LP, College Station, USA) for all analyses.

Kruskal–Wallis tests or contingence tables were used to assess BMI-specific differences in continuous and categorical variables, respectively. Spearman correlation coefficients were provided to show simple relationships. Number of natural teeth was used as a continuous variable or stratified into quartiles comprising 0–18, 19–23, 24–26 and 27–28 teeth. We focused on the quartiles of natural teeth number to divide the population into four equal parts with identical numbers of participants. This approach is a robust method that makes no distributional assumption about the error term and is insensitive to outliers. Thus, we avoided stratification into uneven groups of tooth loss as generally used when examining for chewing difficulties or for diagnosis of oral dysfunction. Multiple linear regression analyses were applied to estimate the effects of chewing muscles and HGS on adiposity figures such as WHR or different fat depots. All estimates were provided with 95% confidence intervals or bands.

## Results

### Baseline data

In this study, 31.5% of participants were of normal weight according to the standard BMI criteria, 43.8% overweight, and 24.7% were obese. Overweight and obese participants were different from their leaner counterparts in all characteristics relevant to this study (Table 1). Dose–response pattern was observed across the BMI categories from normal weight to overweight to obesity for fat distribution, chewing muscle extent, periodontal probing depth, number of teeth, and inflammatory markers such as CRP and fibrinogen. Relative hand grip strength expressed as HGS/BMI also followed this pattern.

**Table 1:** Baseline characteristics of the participants by BMI categories (*N* = 616)

	BMI normal (*N* = 194)	Overweight (*N* = 270)	Obese (*N* = 152)	*p*
Age, years	52.7 ± 12.8	57.2 ± 12.6	56.4 ± 11.6	< 0.001
Female participants (%)	137 (70.6)	106 (39.3)	73 (48.0)	< 0.001
Body mass index BMI, kg/m^2^	23.0 ± 1.5	27.2 ± 1.4	32.9 ± 2.8	< 0.001
M. masseter area, cm^2^	4.0 ± 1.0	4.4 ± 1.1	4.6 ± 1.1	< 0.001
M. pterygoideus medialis area, cm^2^	2.2 ± 0.5	2.5 ± 0.6	2.6 ± 0.5	< 0.001
M. pterygoideus lateralis area, cm^2^	3.1 ± 0.6	3.4 ± 0.6	3.4 ± 0.6	< 0.001
Hand grip strength (HGS), kg—male	46.9 ± 8.7	46.3 ± 9.0	46.4 ± 9.0	0.99
Female	27.2 ± 5.5	27.3 ± 5.7	28.0 ± 5.5	0.23
HGS/BMI^a^—male	1.98 ± 0.36	1.70 ± 0.34	1.44 ± 0.29	< 0.001
Female	1.21 ± 0.27	1.02 ± 0.22	0.85 ± 0.18	< 0.001
Waist-to-hip ratio (WHR)—male	0.90 ± 0.05	0.94 ± 0.04	0.99 ± 0.05	< 0.001
female	0.80 ± 0.05	0.83 ± 0.05	0.86 ± 0.05	< 0.001
BIA body fat, in percent—male	20.1 ± 4.6	23.3 ± 4.1	27.2 ± 4.1	< 0.001
Female	28.2 ± 4.0	34.6 ± 3.2	40.8 ± 3.8	< 0.001
Subcutaneous fat, Liter—male	4.4 ± 1.2	6.1 ± 1.5	9.3 ± 2.5	< 0.001
Female	5.8 ± 1.6	8.8 ± 1.4	13.3 ± 3.1	< 0.001
Visceral fat, Liter—male	3.5 ± 1.5	5.3 ± 1.8	8.2 ± 2.2	< 0.001
Female	1.7 ± 0.9	3.0 ± 1.3	4.7 ± 1.6	< 0.001
No. of teeth, mean (0–28)	21.7 ± 7.0	20.2 ± 8.1	20.2 ± 7.3	< 0.001
Mobile dentures, *N* (%)	34 (17.6)	68 (25.3)	37 (24.5)	0.13
Mean PD, mm	2.5 ± 0.6	2.7 ± 0.6	2.7 ± 0.6	< 0.001
Mean CRP, mg/L (max. 10.0)	1.1 ± 1.0	1.6 ± 1.5	2.6 ± 2.3	< 0.001
HbA1c, %	5.1 ± 0.6	5.4 ± 0.6	5.8 ± 1.1	< 0.001
Smoking, current	32 (16.5)	44 (16.3)	29 (19.1)	0.75
Education, less than 10th grade	30 (15.5)	65 (24.1)	30 (19.7)	0.074
Physical activity (yes), *N* (%)	110 (56.7)	132 (48.9)	63 (41.5)	0.018

Data are presented as numbers (percent), mean ± SD

^a^Relative hand grip strength in kg/BMI

The correlation matrix of the variables of interest, namely age, number of teeth, masticatory muscle, HGS, BMI, WHR, and body fat (in % of weight), is given in Table 2. The number of teeth was inversely associated with WHR and body fat but positively associated with BMI. Masseter muscle was positively associated with BMI and WHR but inversely associated with the percentage of body fat. Similarly as shown with the masseter, also HGS was positively associated with BMI and WHR. There exists a strong correlation between HGS and masseter muscle (*p* < 0.001). With increased age, the number of teeth decreased, whereas HGS, and masticatory muscle areas weakened with age. All adiposity figures worsened with age.

**Table 2:** Correlation matrix of main variables of interest, Spearman’s $\rho$

	Age	No. of teeth	Masseter	HGS	BMI	WHR
Age, years	1.000					
Number of teeth	– 0.591	1.000				
Masseter area, cm^2^	– 0.212	0.373	1.000			
HGS, kg	– 0.224	0.185	0.555	1.000		
BMI, kg/m^2^	0.185	0.144	0.198	0.109	1.000	
WHR	0.235	– 0.133	0.417	0.548	0.444	1.000
BIA body fat, %	0.022	– 0.088	– 0.347	– 0.555	0.447	– 0.297

*All significant at *p* < 0.001 except body fat vs. age (*p* = 0.55), body fat vs. teeth (*p* = 0.018), and BMI vs. hand grip strength (*p* = 0.004)

**Fig. 1:**
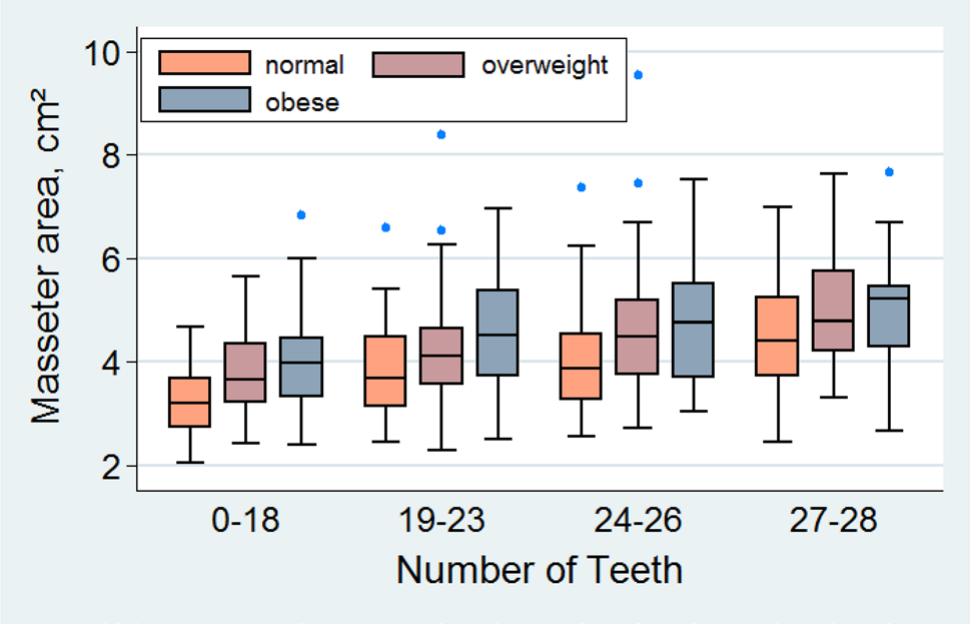
Distribution of masseter muscle areas by BMI categories and number of teeth categorised in quartiles

Masseter muscle areas were more expanded with an increased number of teeth and its area was greater the higher the BMI was in each of the tooth quartiles as it is shown in the box plot depicted in Fig. 1. As shown in Table 3, the masseter area was significantly associated with HGS, irrespective of whether teeth number was included as an independent variable or not. There was a clear dose–response relation by the number of natural teeth on masseter. Masseter muscle area was positively associated not only with the number of teeth but also negatively associated with periodontal disease as expressed by PD. Analogous associations were observed with the pterygoids except with the number of teeth, which were of minor impact (Table S1). To compare the areas of the chewing muscles examined, the cumulative area distributions of all three, the masseter and both pterygoids are supplemented in Fig. S1.

**Table 3:** Relationship between masseter muscle area (cm^2^, dependent) and hand grip strength without and with the influence of dentition tooth status

	Analysis without teeth *ß* (95% CI)	*p*	Analysis with teeth ß (95% CI)	*p*
Hand grip strength, 5 kg	0.07 (0.02; 0.12)	0.008	0.07 (0.02; 0.12)	0.008
BMI, kg/m^2^	0.04 (0.03; 0.06)	< 0.001	0.04 (0.03; 0.06)	< 0.001
HbA1c, %	0.06 (– 0.04; 0.16)	0.22	0.04 (– 0.06; 0.14)	0.41
Age, years	– 0.01 (– 0.02; – 0.00)	0.005	– 0.00 (– 0.01; 0.00)	0.37
Female sex (ref. male)	– 0.87 (– 1.12; – 0.62)	< 0.001	– 0.83 (– 1.08; – 0.59)	< 0.001
Probing depth, mm	– 0.24 (– 0.36; – 0.12)	< 0.001	– 0.18 (– 0.31; – 0.06)	0.004
Mobile dentures (yes/no)	– 0.48 (– 0.67; – 0.28)	< 0.001	– 0.23 (– 0.47; 0.01)	0.064
Number of teeth, 0–18	–	–	0	Ref
-, 19–23	–	–	0.25 (0.01; 0.49)	0.043
-, 24–26	–	–	0.40 (0.13; 0.66)	0.003
-, 27–28	–	–	0.72 (0.44; 1.01)	< 0.001

Additionally adjusted for smoking (never, quitted, current), education (less than 10th grade, yes/no), physical activity (yes/no), reduced to *N* = 571 due to lacking probing depth data

### Regression of adiposity against muscles

Table 4 presents body fat mass regressed on masseter muscle or HGS as unique independents or, combined, adjusted for relevant covariates. Both masseter and HGS remained inversely associated with body fat even after adjustment to each other. HGS attenuated the masseter coefficient by 4%, but inserting the masseter area attenuated the HGS coefficient by 22%. Considerable sex differences were obvious, and thus, we stratified the analysis given in Table 4 by sex and collected the results in the supplement Table S2. The sex differences observed were especially related to HGS and masseter, which are generally known as inevitably different between men and women. It should be noted that these inverse associations shown were only significant when adjusted for BMI (or body weight for that matter).

**Table 4:** Regression of BIA-assessed body fat percentage against hand grip strength (HGS) and/or masseter muscle (*N* = 616)

Independents^†^	Masseter *ß* (95% CI)	Grip Strength *ß* (95% CI)	Combined *ß* (95% CI)
HGS, 5 kg steps	–	– 0.25 (– 0.47; – 0.02)*	– 0.20 (– 0.42; 0.02)
Masseter area, cm^2^	– 0.79 (– 1.15; – 0.44)**	–	– 0.76 (– 1.12; – 0.41)**
Number of teeth, 0–18	Ref	Ref	Ref
-, 19–23	0.42 (– 0.62; 1.47)	0.19 (– 0.86; 1.24)	0.43 (– 0.62; 1.48)
-, 24–26	0.40 (– 0.74; 1.55)	0.14 (– 1.01; 1.29)	0.50 (– 0.65; 1.65)
-, 27–28	0.18 (– 1.08; 1.43)	– 0.43 (– 1.68; 0.81)	0.18 (– 1.08; 1.44)
Wearing mobile dentures	0.23 (– 0.81; 1.28)	0.45 (– 0.60; 1.51)	0.24 (– 0.81; 1.28)
HbA1c, %	0.12 (– 0.29; 0.53)	0.09 (– 0.32; 0.51)	0.13 (– 0.29; 0.54)
BMI, kg/m^2^	1.07 (0.99; 1.14)**	1.03 (0.95; 1.11)**	1.06 (0.99; 1.13)**
Age, years	– 0.03 (– 0.06; 0.01)	– 0.03 (– 0.07; 0.00)	– 0.04 (– 0.07; – 0.00)*
Female sex (ref. male)	9.71 (8.99; 10.4)**	9.60 (8.53; 10.7)**	8.98 (7.87; 10.1)**

^†^Additionally adjusted for smoking (never, quitted, current), education (less than 10th grade, yes/no), physical activity (yes/no), *p < 0.05, **p < 0.001

### Different fat depots

**Fig. 2:**
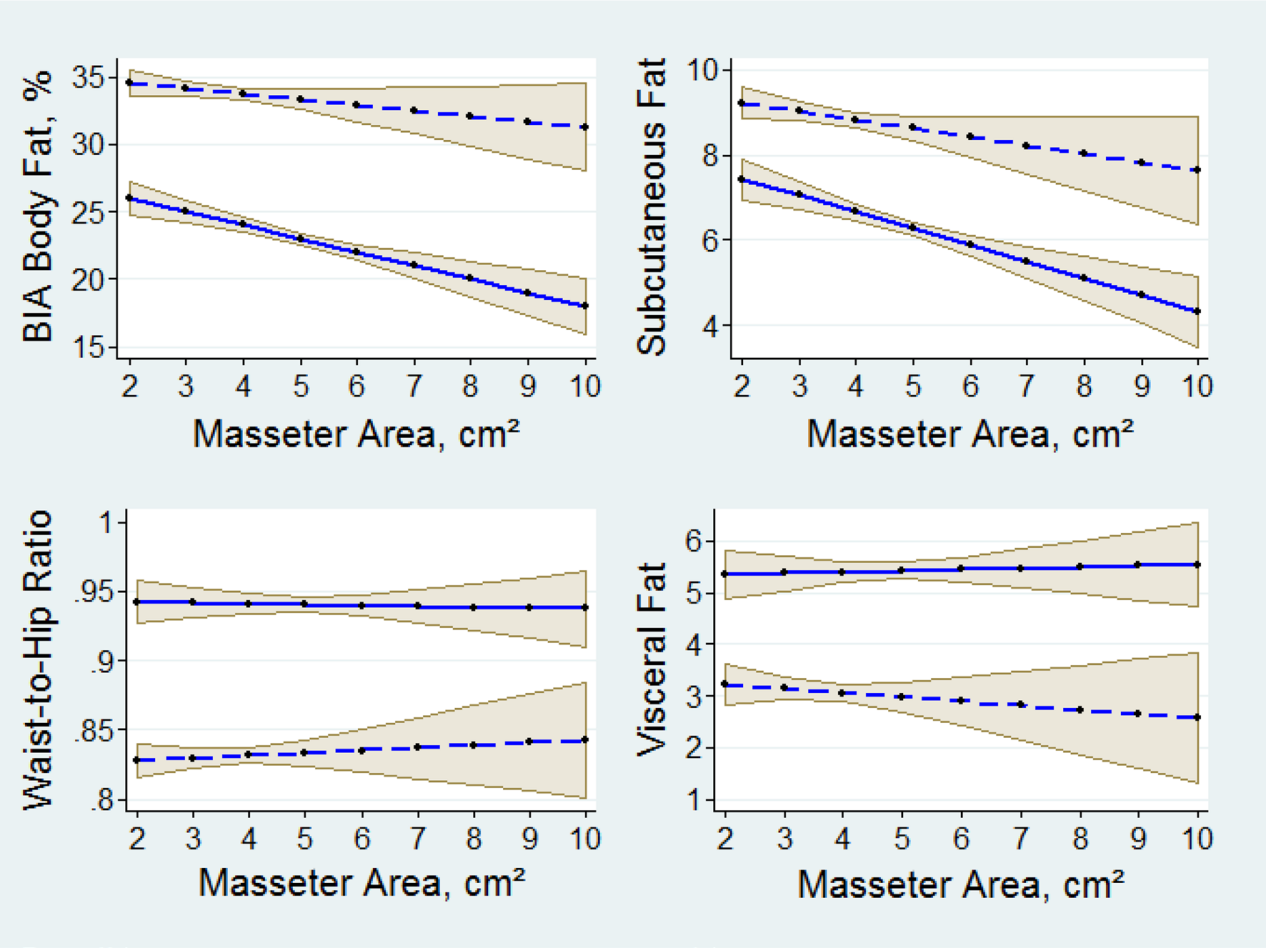
Predictive margins of adiposity measures by masseter muscle area. Solid lines men, dashed lines women, shaded areas are 95% confidence bands (*N* = 616)

MRI measurements of fat depots were available. To compare the different relationships between adiposity and chewing muscles, in Fig. 2 correlations are displayed between the adiposity parameters analyzed and the masseter muscle area, each adjusted to age, teeth and BMI after multiple regression. While revealing typical sex differences, BIA-derived body fat and subcutaneous fat appeared inversely associated with masseter area indicated by a downward slope (Fig. 2). Masseter area was significantly associated with subcutaneous fat in both men and women. In contrast, with WHR and visceral fat as dependents, the masseter seemed to be without such influence.

## Discussion

The inter-relationship of food processing between teeth and chewing muscles is very complex, but more so are the associations of oral capabilities with obesity. First, there is a reasonable presumption that a bidirectional relationship exists between obesity and oral conditions [3] and, secondly, all relevant factors are age-dependent and it is quite cumbersome to differentiate if there are true inter-dependencies or merely concurrent processes associated with ageing [19]. Ageing is related to different pathophysiological changes accompanied by reductions in muscle mass and strength.

In this cross-sectional and population-based study, we observed that the chewing muscles masseter and the pterygoids are closely related to hand grip strength indicating that the chewing muscles’ strength follows that of the general muscular system. The state of this system may be a proxy of the general health state reflected here by the masseter as it is true for HGS. The cross-sectional area of these muscles is related to their muscle strength and the bite force [16]. Though teeth are the main determinant of the masseter’s strength, they do not alter the relationship between HGS and the masseter area. In agreement with our results, Yamaguchi and co-workers reported that tooth loss was more important than age for loss of masseter thickness and HGS remained independently related to the masseter, particularly in men [6]. Age and teeth number are closely correlated. Notwithstanding, as compared to teeth as determinants of masticatory muscles, age was of minor influence on the masseter or the relation between masseter and obesity (Tables 2, 3). Others also observed that age and sex did not show a strong effect on masticatory performance as assessed through masseter cross-sectional area notwithstanding that the muscles tend to decline with age [20]. As masseter muscles’ strength is determined by the number of functional teeth, so is also HGA positively associated with the number of teeth [21].

Interweaved mutual relationships exist between physical fitness, general health state, mastication and obesity in the context of age-related loss of functions. This raises obstacles to disentangling the network for single causal pathways [3, 6, 22]. The health consequences associated with pathological adiposity states are complex as they are modified in parallel by both ageing and obesity [23]. Decline in muscle mass by increasing age and infiltration of muscular tissue by fat cells may contribute to muscle disease and lead to sarcopenia, which possibly also affects the chewing musculatures [5]. Certainly, the association between tooth loss and obesity was independent of differences in age and gender as reported in a Swedish study with participants below 60 years of age [24].

In our study, both HGS and masseter were associated with BIA fat mass and subcutaneous fat. In regression analyses the HGS effect was partially attenuated when adjusted additionally for masseter muscle, indicating partial independence of each other. For WHR and visceral fat no such relationship was observed. This difference was unexpected and remains unexplained as visceral fat depots are expected to be the metabolically more active fat tissue [25]. In crude correlations, WHR was positively associated with masseter and HGS but BIA body fat negatively (Table 2). In our data visceral fat was positively associated with masseter and HGS, but subcutaneous fat was negatively associated. However, in a huge English population study, Keevil and coworkers reported decreasing HGS with increasing WC in all BMI categories [26]. Both obesity and periodontitis-mediated tooth loss are characterized by a low-grade inflammatory state. This is considered to be related to the metabolically active adipose tissue found in the visceral region. So are cardiometabolic risk factors more strongly associated with visceral than with subcutaneous fat [27].

There are two opposing directions of the association between mastication and obesity. It was shown that obese persons suffered from changes in masticatory behavior [28]. Altered food choices and a sedentary lifestyle may induce a self-enforcing circle between obesity and food intake. Even in people with a full set of natural teeth, chewing behaviour is associated with body weight [29]. On the other hand, the declining masticatory ability associated with ageing and age-related oral deteriorations such as periodontitis and tooth loss are considered causative for obesity. According to NHANES data, especially central obesity was associated with tooth loss even in a non-obese population [2]. Tooth loss is associated with reduced performance of chewing muscles [14] leading to impaired chewing efficiency [3] altogether eventually resulting in overweight or even obesity [1]. In a recent review, the authors reckon that poor oral health with a lower number of teeth is inversely associated with the intake of vegetables, fruits and dietary fibres leading to sarcopenia [8]. Accordingly, total loss of teeth, i.e. edentulism, is associated with obesity [7]. Thus, maintaining proper bite force and masticatory capability are important factors in maintaining normal body weight and shape [30]. Inversely, some studies identified obesity as a risk factor for tooth loss, whereby the chronic generalized inflammation that is generated by a combination of factors in obese individuals reduces the immune threshold, making them more susceptible to periodontal disease, a major reason for tooth loss [1, 21, 30].

Some limitations should be noted. This was a cross-sectional study, therefore no cause-effect relations could be determined preventing an ultimate conclusion. The number of teeth was used instead of the number of functional tooth units, which would report regions of occlusal support and, therefore, being closer to the true chewing ability. Although this analysis was part of a population study, there was a selection bias insofar as from the original baseline study SHIP-START-0 predominantly the very old or sick participants dropped out and were no longer available 10 years later in SHIP-START-2 examined here. While tooth loss rates have decreased globally [31], the prevalence of obesity has risen considerably in the past 40 years. The increase is probably due to changes in the relationship between eating behaviour, weight, body fat, and eating behaviour partially directed by oral conditions [32].

To conclude, masticatory muscles, dentition, and physical fitness are interweaved in such a way that their interplay is markedly associated with body shape. If confirmed in longitudinal studies, then it must be accepted that muscle-controlled physical fitness and masticatory performance are important for maintaining a healthy body shape. There is a possible clinical implication, especially for general dental practice. The recommendation for physical activity or training, beyond its positive effects boosting overall fitness, may improve masticatory performance as well.

## Data Availability

Data from SHIP are available after data application and signature of a data transfer agreement. The data dictionary and the online application form are available at: http://fvcm.med.uni-greifswald.de/dd_service/data_use_intro.php. Involving a local collaborative partner to facilitate the application process is recommended.
